# Editorial: Overcoming challenges in mucosal immunity: 2022

**DOI:** 10.3389/fimmu.2023.1327091

**Published:** 2023-11-07

**Authors:** Javier Leceta, Jorge Galindo-Villegas

**Affiliations:** ^1^ Department of Cell Biology, Faculty of Biology, Complutense University of Madrid, Madrid, Spain; ^2^ Department of Genomics, Faculty of Biosciences and Aquaculture, Nord University, Bodø, Norway

**Keywords:** 3-hydroxy-3-methylglutaryl-CoA synthase 2, A20, immunonutrition, immunosenescence, unfolded protein response

The mucous-producing membranes house the highest concentration of lymphoid tissue in the vertebrate body due to their extensive interaction with the external environment. Although considerable research effort has been devoted to their study, certain crucial aspects necessitate innovative investigative approaches. Preserving mucosal integrity and regulating immune responses, both locally and systemically, are critical for maintaining homeostasis. This Research Topic is dedicated to addressing fundamental questions pertaining to mucosal immunity and its associated challenges. These challenges encompass aspects such as the role of the epithelium in governing both mucosal and systemic immune responses. Contributions to this Research Topic shed light on various topics, for example, the preservation of mucosal barrier integrity, the influence of nutritional elements, namely amino acids, short-chain fatty acid (SCFA) metabolism, and the unfolded protein response (UPR) within intestinal epithelial cells (IEC). Additionally, this Research Topic delves into immunological senescence and its relevance to mucosal immunity, particularly in the context of aging populations.

The study by Ouyang et al. focused on patients with HIV infection undergoing antiretroviral therapy. Despite the reduction in morbidity and mortality rates in treated patients, their immune systems do not appear to be fully restored, leading to chronic inflammatory infections and damage to the intestinal epithelial barrier. This review explored the feasibility of using liquid blood biomarkers as indicators of intestinal barrier damage in HIV patients, as invasive intestinal biopsies are impractical for this purpose. Damage to the intestinal mucosal barrier allows microbial products to translocate into the systemic circulation, contributing to chronic inflammation and elevating the risk of non-AIDS-related health issues. Therefore, the study provided insight into plasma biomarkers of intestinal permeability and envisions their future application in therapeutic strategies to address leaky gut in individuals living with HIV.

Martín-Adrados et al. investigated the unfolded protein response (UPR) in intestinal epithelial cells, a response associated with inflammatory bowel disease. Inflammatory triggers prompt intestinal epithelial cells to produce excessive amounts of proteins, rendering them susceptible to the UPR, which, in turn, leads to severe inflammatory conditions. In this study, the researchers identified several UPR-regulated genes in IECs, including the enzyme 3-hydroxy-3-methylglutaryl-CoA synthase 2 (HMGCS2), which plays a role in short-chain fatty acid (SCFA) metabolism. However, the use of butyrate as a treatment for ulcerative colitis has yielded variable outcomes, with some patients benefiting more than others. As a result of this research, the authors suggest that determining HMGCS2 expression levels may serve as a marker for patients who could potentially benefit from controlled dietary butyrate supplementation.

The article by Hissen et al. introduced the concept of immunonutrition as a modulator of the immune response in vertebrates, particularly in the context of gut mucosal immunity, and from a comparative perspective. The study initially outlined the distinct characteristics of the immune response in teleost fishes, focusing on innate molecular mechanisms related to pathogen-associated recognition, the expression of inflammatory cytokines, the complement system, respiratory burst, and the generation of oxygen and nitric oxide free radicals. The authors emphasized the importance of dietary amino acid proportions, underscoring the need to provide specific essential/functional amino acids and discussing the potential of amino acid supplementation. Furthermore, the authors analyzed the immunomodulatory effects of glutamine, alpha-ketoglutaric acid, and arginine, highlighting their roles in oxidative homeostasis, maintenance of gut epithelial barrier integrity, and nitric oxide production, respectively. The study recommends expanding the research using genomic and proteomic tools, cell culture, and cross-species investigations, recognizing the differences among species.

Another essential aspect of the immune response involves the regulation necessary to maintain homeostasis. Dysregulation of immune inhibitory mechanisms within mucous membranes can lead to hyper-inflammatory states, potentially resulting in allergic diseases. A20, or TNF-α-induced protein 3 (TNFAIP3), an endogenous master regulator with anti-inflammatory properties and ubiquitin-modifying enzyme activity that inhibits NF-κB signaling, was the focus of the review by Liu et al. Their study summarized the role of A20 in allergic processes in the respiratory tract, elucidating the cellular and molecular mechanisms that govern its signaling.

The research by Allen et al. delved into the topic of mucosal immunosenescence and the systemic response to an attenuated oral *Salmonella typhimurium* vaccine. While young adult mice exhibit lower *Salmonella* levels in various organs, older individuals do not show the same trend. Aged mice also display reduced *Salmonella*-specific antibody titers in both serum and feces following immunization. Given that salmonellosis is the leading cause of hospitalization due to foodborne enteric infections, primarily affecting individuals aged 65 and older, these findings have implications for vaccine development in older populations and underscore the urgent need for a more comprehensive understanding of immunosenescence mechanisms in mucous membranes.

In summary, this Research Topic has explored the intricate world of mucosal immunity and emphasized its importance due to the extensive interaction of mucosal membranes with the external environment. Despite significant research efforts, there remain challenging aspects for the next few years that will demand innovative investigative approaches to advance our understanding of this critical area. Preserving the integrity of mucosal barriers and orchestrating immune responses, both locally and systemically, are central to maintaining overall homeostasis. The contributions in this Research Topic illuminate various facets of mucosal immunity, addressing topics such as mucosal barrier integrity, nutritional factors like amino acid and short-chain fatty acid metabolism, and the unfolded protein response (UPR) in intestinal epithelial cells (IEC). Moreover, the research extends its reach to explore immunological senescence within the context of mucosal immunity, particularly in the older population, shedding light on the multifaceted interplay between aging and immune responses. Several studies highlight the implications of these findings in various health contexts, such as chronic inflammatory infections in HIV patients, the potential markers of butyrate treatment efficacy in ulcerative colitis, the immunomodulatory effects of amino acids, and the complex role of A20 in regulating allergic processes in the respiratory tract. Additionally, the study of mucosal immunosenescence and its impact on systemic responses to *Salmonella* vaccines in elderly populations provides crucial insights for future vaccine development. Altogether, these contributions reinforce the importance of in-depth research into mucosal immunity and its multifaceted connections to various health-related issues and underscore the need for continued exploration and understanding in this field.

## Author contributions

JL: Writing – original draft. JG: Writing – review & editing.

